# Editorial: Chemo-Radiation-Resistance in Cancer Therapy

**DOI:** 10.3389/fphar.2022.904063

**Published:** 2022-05-18

**Authors:** Xiaoping Lin, Dexin Kong, Zhe-Sheng Chen

**Affiliations:** ^1^ State Key Laboratory of Oncology in South China, Department of Nuclear Medicine, Collaborative Innovation Center for Cancer Medicine, Sun Yat-Sen University Cancer Center, Guangzhou, China; ^2^ Tianjin Key Laboratory on Technologies Enabling Development of Clinical Therapeutics and Diagnostics, School of Pharmacy, Tianjin Medical University, Tianjin, China; ^3^ Department of Pharmaceutical Sciences, College of Pharmacy and Health Sciences, Institute for Biotechnology, St. John’s University, Queens, New York, NY, United States

**Keywords:** chemothearpy resistance, radiationtherapy resistance, cancer, chemoradiation resistance, therapy

In recent years, technical advances in chemotherapy and radiotherapy have helped substantially improve the treatment outcome and quality of life of cancer patients. Nevertheless, successful cancer therapy remains a major challenge, particularly in tumors that are resistant to chemotherapy or radiation therapy. Searching the topic “Chemoradiation Resistance in Cancer Therapy” results in 34 articles (six reviews, 27 original research, and one brief research report) contributed by more than 262 authors with over 90000 views in all of time until 20 April 2022, in the fields of cancer diagnosis and therapeutics. Our aim was to generate a collaborative discussion contributing to the future direction of overcoming chemoradiation resistance and improve cancer patient care during chemo- and/or radiation therapy.

Characteristics of chemo-and radiation-resistant cells include altered membrane transporter expressions and functions, enhanced DNA repair activity, apoptotic pathway defects, alteration of target molecules, or enzymatic deactivation. There are two general causes of failure of antineoplastic therapy: Inherent genetic characteristics that induce resistance in cancer cells and acquired resistance after drug exposure and radiation exposure. As the primary anti-cancer therapies, ionizing radiation and chemotherapeutic agents induce cell death by directly or indirectly causing DNA damage, dysregulation of the DNA damage response may contribute to hypersensitivity or resistance of cancer cells to genotoxic agents. Targeting DNA repair pathway can therefore increase the tumor sensitivity to cancer therapies. While more attention have been paid lately to the relationship between defective nuclear DNA repair pathway and therapeutic resistance, less is known about the role of mitochondrial repair pathways. Lan-Ya Li et al. (Li et al., 2021) reviewed the biology and the regulatory mechanisms of DNA repair pathways, which has the potential to facilitate the development of inhibitors of nuclear and mitochondria DNA repair pathways for enhancing anticancer effect of DNA damage-based therapy.

Platinum resistance poses a significant problem for oncology clinicians. The role of epigenetics and DNA methylation in platinum-based chemoresistance has gained increasing attention from researchers in recent years. Ruizheng Sun et al. (Sun et al., 2021) analyzed the platinum chemotherapy response-related methylation patterns from different perspectives of 618 patients across 13 cancer types and integrated transcriptional and clinical data. They indicated that the methylation-transcription axis exists and participates in the complex biological mechanism of platinum resistance in various cancers. Six methylated positions (differentially methylated positions, DMPs) and four associated genes may have the potential to serve as promising epigenetic biomarkers for platinum-based chemotherapy and guide clinical selection of optimal treatment.

Cisplatin (DDP) is commonly used for gastric cancer treatment, whereas recurrence and metastasis are common because of intrinsic and acquired DDP-resistance. Experiments by Yingying Kou et al. (Kou et al., 2020) suggested that berberine improves chemo-sensitivity to cisplatin by enhancing cell apoptosis and repressing PI3K/AKT/mTOR signaling pathway.

Cisplatin-based regimens also commonly applied for nasopharyngeal carcinoma (NPC) patients receiving concurrent chemotherapy and radiation. The sensitivity of cisplatin is closely associated with the efficacy of radiation therapy. Wenwen Hao et al. (Hao et al., 2021) found that Solute Carrier Family 1 Member 6 (SLC1A6) contributed to reducing cisplatin sensitivity in radioresistant NPC cells by altering drug metabolism profiles and genes.

Carboplatin is the cornerstone of chemotherapy for ovarian cancer. However, drug resistance to this agent continues to present challenges, and the mechanism of resistance to carboplatin in ovarian cancer has become a focus of research in recent years. Increasing evidence has shown that collagen type I alpha 1 chain (COL1A1) has an important role in chemoresistance and could represent a potential therapeutic target, but the mechanism of COL1A1 in carboplatin-resistant ovarian cancer has remained unclear. Feng Yang et al. (Yang et al., 2021) discovered that COL1A1 had a pivotal role in carboplatin resistance in ovarian cancer and identified two key pathways involving COL1A1 in carboplatin resistance: the “extracellular matrix (ECM)-receptor interaction” and “focal adhesion” Kyoto Encyclopedia of Genes and Genomes pathways. Furthermore, they proposed that ZINC000085537017 and quercetin were potential drugs for COL1A1 based on virtual screening and the TCMSP database, respectively.

Besides platinum, paclitaxel (PTX) is a first-line chemotherapeutic drug for the treatment in many different types of cancer, but drug resistance seriously limits its clinical use. Fenfen Xiang et al. (Xiang et al., 2021) showed that DNA-methyltransferase 1 (DNMT1) mediated hypermethylation of Krüppel-like factor 4 (KLF4) promoter leads to downregulation of KLF4 in breast cancer. The level of KLF4 is correlated with the sensitivity of MCF-7 and T47D cells to PTX 3,3′-diindolylmethane (DIM) could enhance the antitumor efficacy of PTX on MCF-7 and T47D cells by regulating DNMT1 and KLF4. In ovarian cancer, Yaqing Zhang et al. (Zhang et al., 2021) found that the drug resistance protein CSAG2 is translationally induced by cytoplasmic polyadenylation element binding protein 4 (CPEB4), which underlies CPEB4-promoted paclitaxel resistance in ovarian cancer *in vitro*. They found interfering CPEB4/CSAG2 axis might be of benefit to overcome paclitaxel-resistant ovarian cancer.

Doxorubicin (DOX) is a first-line chemotherapeutic drug for breast cancer, which can kill tumor cells but it causes multidrug resistance (MDR) if used for a long period of time, resulting in chemotherapy failure. In DOX-resistant breast cancer cells, P- glycoprotein protein can pump DOX out of MCF-7/DOX cells, as a result, DOX fails to exert effective cytotoxic effect and breast cancer cells can evade attack of chemotherapeutics. Reversal of drug resistance can be realized by repressing P- glycoprotein protein. Ting Wang et al. (Wang et al., 2021a) identified that a new chalcone derivative, C49, reverses DOX resistance in MCF-7/DOX cells by inhibiting P-glycoprotein expression.

Despite chemotherapy is the most effective treatment for breast cancer, many patients develop chemoresistance. Early indicator of therapy efficacy might aid in the search for better treatment and patient survival. Emerging evidence indicates a key role of the purinergic receptors P2X7 and A2A in cancer. Victor Manuel Ruiz-Rodríguez (Ruiz-Rodríguez et al., 2020) explored the purinergic receptors P2X7 and A2A in cancer and their involvement in breast cancer chemoresistance, demonstrating the importance of purinergic signaling in CD8^+^ T cells during chemoresistance as the chemotherapeutic treatment stimulates immune system response, and how it could be considered for implementing personalized therapeutic strategies.

Both long-term anti-estrogen therapy and estrogen receptor-negative breast cancer contribute to drug resistance, causing poor prognosis in breast cancer patients. Breast cancer resistance protein (BCRP) plays an important role in multidrug resistance. The study by Wenting Ni et al. (Ni et al., 2021) suggested that cryptotanshinone (CPT) is a novel BCRP inhibitor that blocks the oligomer formation of BCRP on the cell membrane. CPT can inhibit the activity of BCRP in an ERα-dependent and -independent manner, sensitizing breast cancer cells to chemotherapy.

Histone deacetylases and histone acetylases (HDACs) are important enzymes participating in the regulation of gene expression by acetylating and deacetylating of histones. Specifically, HDACs are the enzymes controlling the epigenetic modifications of histone. In recent years, inhibition of HDACs has exhibited potency for the treatment tumors. Nitrogen mustard anticancer drugs were used clinically since 1942, which effectively bind and cross-link to DNA, resulting in prevention of DNA replication and cell proliferation. Yiming Chen et al. (Chen et al., 2020b) discovered that N-(2-amino-4-fluorophenyl)-4-[bis-(2-chloroethyl)-amino]-benzamide (FNA) was a potent HDAC3 inhibitor by inhibiting tumor growth and promoting apoptosis and G2/M phase arrest, which improved the anticancer activity of paclitaxel and camptothecin.

Overexpression of nucleophosmin (NPM) is involved in the MDR development during acute lymphoblastic leukemia (ALL). Donghui Gan et al. (Gan et al., 2021) identified that doxorubicin/nucleophosmin binding protein-conjugated nanoparticle (DOX-PMs-NPMBP) was able to significantly exert growth inhibition and apoptosis induction, and markedly enhance anti-leukemia activity in acute lymphoblastic leukemia cells *in vitro* and *in vivo* . Mechanistically, p53-driven apoptosis induction and cell cycle arrest played essential role in DOX-PMs-NPMBP-induced anti-leukemia effects.

Nowadays, many natural-derived drugs serve as sources of novel drug discovery and are tested clinically. However, the efficacy of certain natural products could be compromised by MDR-associated ATP-binding cassette (ABC) transporters. ABC subfamily C member 1 (ABCC1, multidrug resistance protein 1/MRP1), ABC sub-family B member 1 (ABCB1, multidrug resistance protein 1/MDR1, P-glycoprotein/P-gp), and ABC sub-family G member 2(ABCG2, breast cancer resistance protein/BCRP, mitoxantrone-resistant protein/MXR) are extensively studies, and are commonly responsible for MDR. Betulin is susceptible to drug resistance mediated by ABCC1 overexpression, and a known ABCC1 inhibitor, MK571, can sensitize the cells expressing ABCC1 to betulin. Xuan-Yu Chen et al. (Chen et al., 2021) explored ABCC1-induced resistance to betulin by its upregulated protein expression of ABCC1 and found that betulin at high concentration had the ability to inhibit ABCC1 transport function, which may affect the pharmacokinetic profile of other ABCC1 drug substrates, such as vincristine.

Sorafenib, a multireceptor tyrosine kinase inhibitor is FDA approved first-line drug for the treatment of advanced liver cancer and is reported to extend the overall survival in individuals with advanced hepatocellular carcinoma (HCC). However, the primary or acquired resistance to sorafenib is gradually increasing, leading to failure of HCC treatment with sorafenib. Wubin He et al. (He et al., 2021) reported that artesunate regulates neurite outgrowth inhibitor protein B receptor (NgBR) to overcome resistance to sorafenib of HCC in a cell culture model.

Typically, renal cell carcinoma (RCC) is insensitive to traditional chemo- and radio-therapeutic treatments. Moreover, the use of targeted treatment options as first- and second-line treatments have limited effect on the survival rates. Dian Fu et al. (Fu et al., 2020) explored low-toxicity novel treatment strategies for RCC and investigated costunolide (Cos), a natural sesquiterpene compound isolated from various medicinal plants, and found that it exerted autophagic and apoptotic effects on renal cancer through the ROS/JNK-dependent signal route.

Chemoresistance has become a prevalent phenomenon in cancer therapy, which alleviates the effect of chemotherapy and makes it difficult to break the bottleneck of the survival rate of tumor patients. Jin-Feng Xu et al. (Xu et al., 2021) reviewed the functional roles of ginsenosides in chemoresistance reversal. Its underlying mechanism is correlated with inhibition of drug transporters, induction of apoptosis, and modulation of the tumor microenvironment(TME), as well as the modulation of signaling pathways, such as nuclear factor erythroid-2 related factor 2 (NRF2)/AKT, lncRNA cancer susceptibility candidate 2(CASC2)/protein tyrosine phosphatase gene (PTEN), AKT/sirtuin1(SIRT1), epidermal growth factor receptor (EGFR)/PI3K/AKT, PI3K/AKT/mTOR and nuclear factor-κB (NF-κB).

Modulated electro-hyperthermia (mEHT), induced by 13.56 MHz radiofrequency, has been demonstrated both in preclinical and clinical studies to efficiently induce tumor damage and complement other treatment modalities. Tamás Vancsik et al. (Vancsik et al., 2021) used a mouse xenograft model of human melanoma (A2058) to test mEHT (∼42°C), both alone and combined with NK-cell immunotherapy. They found that mEHT monotherapy of melanoma xenograft tumors induced irreversible heat and cell stress leading to caspase-dependent apoptosis to be driven by p53. mEHT could support the intra-tumoral attraction of distantly injected NK cells, contributed by CXCL11 and MMP2 upregulation, resulting in additive tumor destruction and growth inhibition.

Many cancer patients who are treated with chemotherapy and/or radiotherapy eventually become resistant, and acquired resistance accounts for the majority of cases. One of the most well understood mechanisms of chemoresistance is the overexpression of ABC transporters. Zhuo-Xun Wu et al. (Wu et al., 2021) reported how to establish a novel irinotecan-resistant human colon cell line to investigate the underlying mechanism(s) of irinotecan resistance, particularly the overexpression of ABC transporters.

Radiotherapy is recommended as an important and effective method for malignant treatment in about half of cancer patients during clinical treatment. Esophageal squamous cell carcinoma (ESCC) patients who have contraindications for surgery or locally advanced disease have a treatment option through Radiotherapy. However, radioresistance is a major cause of treatment failure, contributing to inadequate cure, relapse, and metastasis. Zuoquan Zhu et al. (Zhu et al., 2021) provided evidence that the FMS-related tyrosine kinase 3 ligand (FL) increases the radioresistance of esophageal cancer cells and that FL-related tyrosine kinase 3 (Flt-3) could be a potential target for enhancing radiosensitivity in ESCC.

More attentions have been attracted to radiosensitizers because of their abilities to increase the radiosensitivity of cancer cells and reduce the side effects on normal cells. In order to identify promising radiosensitivity agents, a large number of natural products with anti-inflammatory, antioxidant, and antitumor activations have been considered. The major treatment modality for non-small-cell lung carcinoma (NSCLC) is radiotherapy. However, radiotherapy can induce radioresistance in cancer cells, thereby resulting in a poor response rate. Yarong Du et al. (Du et al., 2021) demonstrated the effects of isorhamnetin (ISO), which is a naturally occurring radiosensitizer, and its impact on the responsiveness of lung cancer cells to irradiation through IL-13 and the NF-κB signaling pathway.

Protein turnover is a dynamically regulated process influencing many important biological functions, including DNA damage response (DDR), cell cycling, and signaling transductions. Pharmacological intervention of protein turnover offers a new therapeutic window for radiosensitization. Driven by their unique cytotoxic mechanisms, the novel strategies targeting the ubiquitin-proteasome system (UPS) with NEDDylation inhibitors and the PROteolysis TArgeting Chimeras (PROTACs) carry great potential as radiosensitizers to improve the efficacy of radiotherapy. The NEDDylation inhibitor MLN4924 exerts several cytotoxic functions, including DNA damage, cell cycle checkpoints dysregulation, and inhibition of NF-κB, and mTOR pathways. Preclinical studies had validated the efficacy of NEDDylation inhibitors as radiosensitizers. Meanwhile, recent progress in PROTAC technology has shown significant improvements in terms of the cellular permeability and substrate specificity. The PROTACs can selectively recruit key proteins related to radioresistance, such as EGFR, androgen receptor (AR) and estrogen receptor (ER), cyclin-dependent kinases (CDKs), MAP kinase kinase 1 (MEK1), and MEK2, for the cullin-RING E3 ligase (CRL)-mediated polyubiquitin conjugation and subsequent degradation. Shuhua Zheng et al. (Zheng and Tao, 2020) summarized basic and preclinical investigations on NEDDylation inhibitors and PROTACs as radiosensitizers.

Chemotherapy is the backbone of subsequent treatment for patients with lung adenocarcinoma (LUAD) exhibiting radiation resistance, and pemetrexed plays a critical role in this chemotherapy. Yuqing Wang et al. (Wang et al., 2021b) assessed changes in the sensitivity of LUAD cells to pemetrexed under radioresistant circumstances. They showed the much lower efficacy of pemetrexed in radioresistant cells than in parental cells, and that the mechanism of action was the significant downregulation of folate receptor alpha (FRα) by long-term fractionated radiotherapy, which resulted in less cellular pemetrexed accumulation. The activation of FRα by decitabine can sensitize radioresistant LUAD cells to pemetrexed both *in vitro* and in xenografts.

NPC is endemic in southern China and South-East Asia. Radiotherapy is the primary treatment for the non-metastatic disease. Although the local control of NPC can be increased by intensity-modulated radiation therapy (IMRT) rather than conventional radiotherapy, approximately 20% of the patients still present locoregional recurrence following radical IMRT. Tumor recurrence has been recommended to have relationship strong association with radio-resistance. Shan-Shan Guo et al. (Guo et al., 2021b) demonstrated the radioresistant function of angiogenin, as a biomarker that can help identify radio-sensitivity, and showed the clinical prognostic significance of ANG, which could help predict radiosensitivity and stratify high-risk patients or tumor recurrence.

Identifying metastasis-associated genes and finding effective targets is the main strategy to prevent metastasis and improve survival of breast cancer. On the explore of putative tumor suppressor protein, Xin An et al. (An et al., 2020) confirmed that Cavin3 expression is significantly downregulated in breast cancer, and correlated with distant metastasis and worse survival. Cavin3 functions as a metastasis suppressor by downregulating the Akt pathway, which suggests that cavin3 can be a potential prognostic biomarker and a target for breast cancer treatment. Studies by Xin Hua et al. (Hua et al., 2020) suggested that IQ motif-containing GTPase activating protein 3 (IQGAP3), the latest identified member of the IQGAP family, was significantly upregulated in breast cancer cell lines and human tumor tissues at both the mRNA and protein level compared to controls, and the expression was an independent prognostic factor among all 257 breast cancer patients in their cohort. Therefore, IQGAP3 may be a reliable prognostic biomarker in breast cancer.

E3 ubiquitin ligases (E3s) are a large class of proteins, and there are over 800 putative functional E3s. E3s play a crucial role in substrate recognition and catalyze the final step of ubiquitin transfer to specific substrate proteins. The diversity of the set of substrates contributes to the diverse functions of E3s, indicating that E3s could be desirable drug targets. The E3s MDM2, FBWX7, and SKP2 have been well studied and have shown a relationship with drug resistance. Strategies targeting E3s to combat drug resistance include interfering with their activators, degrading the E3s themselves and influencing the interaction between E3s and their substrates. Yuanqi Liu et al. (Liu et al., 2021) summarize the role of E3s in cancer drug resistance from the perspective of drug class and the most important research findings of targeting the cullin-RING E3 ligases for radiosensitization.

The crosstalk between cancer cells and their microenvironment triggers a variety of critical signaling cues and promotes the malignant phenotype of cancer. As a type of transmembrane protein, integrin-mediated cell adhesion is essential in regulating various biological functions of cancer cells. Integrins are the adhesion molecules and receptors of ECM. They mediate the interactions between cells-cells and cells-ECM. Recent evidence has shown that integrins present on tumor cells or tumor-associated stromal cells are involved in ECM remodeling, and as mechanotransducers sensing changes in the biophysical properties of the ECM, which contribute to cancer metastasis, stemness and drug resistance. Chao-Yue Su et al. (Su et al., 2020) outlined the mechanism of integrin-mediated effects on biological changes of cancers and highlight the current status of clinical treatments by targeting integrins.

Most tumor cells are in a hypoxic microenvironment that promotes resistance to radiation therapy. In addition to radiation resistance, the hypoxic microenvironment also promotes cancer proliferation and metastasis. Cordell Gilreath et al. (Gilreath et al., 2021) reviewed the hypoxic microenvironment of breast cancer tumors, related signaling pathways, breast cancer stem-like cells, and the resistance to radiation therapy.

Microenvironmental serine may alter cancer proliferation and invasion. A high serine content in body fluid was identified in a portion of patients with gastric cancer, but its biological significance, such as cell growth, migration and invasion, and drug resistance, was not clearly. Jun Li et al. (Li et al., 2020) characterized the basal gene expressing profiles of MGC803 and HGC27. The HGC27 cells were more differentiated than MGC803 cells while MGC803 cells were more sensitive to the change of serine content. They demonstrated that genetic profiles can affect the biological effects of serine on gastric cancer cells.

The tumor immunological microenvironments of gliomas differ based on their molecular properties. In glioblastoma (GBM), Ji Zhang et al. (Zhang et al., 2020) profiled the immune status of O-6-methylguanine-DNA methyltransferase (MGMT) promoter methylation in GBM and established a local immune signature for GBM that could independently identify patients with a favorable prognosis, indicating a relationship between prognosis and GBM immune signature. MGMT promoter methylation with lower Tim-3 expression was significantly associated with better survival.

Tumorigenesis is strongly associated with a series of cumulative genetic and epigenetic changes occurring in a normal cell; it is also closely related to the body’s microenvironment and immunity. The immune system recognizes and kills cancerous cells and their precursors, while cancerous cells develop strategies to escape from immune-surveillance thereby promoting tumorigenesis. Recently, long non-coding RNA (lncRNA) was proven to play an active part in the regulation of the immune system by affecting tumor microenvironment, epithelial-mesenchymal transition, dendritic cell and myeloid-derived stem cell regulation, and T and B cell activation and differentiation. Immune-related lncRNAs, which were identified as a prognostic marker of various types of cancer, are markedly connected with immune cell infiltration, and might be a potential target for cancer treatment. Peijie Chen et al. (Chen et al., 2020a) constructed a prognostic model and explored the immune characteristics of different risk groups in cervical cancer patients to analyze the relationship between immune-related lncRNAs and the prognosis.

Inherent gene/protein-associated drug and radiation resistance is largely rooted in cancer cell heterogeneity. For this type of resistance, we may be able to detect variants through gene sequencing, flow cytometry, and microarray to determine their mechanisms of resistance, and guide physicians in choosing the right approach for their individual patients. While variants identified for leukemia and lung cancer have improved our ability to predict prognoses and provide personalized medical care, there remain other types of cancer where many new advances could be made.

The efficiency and safety of hypofractionated radiotherapy (HFR) combined with paclitaxel chemotherapy for the treatment of post surgery tracheoesophageal groove lymph node (TGLN) metastasis in patients with esophageal cancer were investigated by Jian Wang et al. (Wang et al., 2020). They found that the combination of hypofractionated radiotherapy (HFR) and chemotherapy improved the prognosis of esophageal cancer patients with tracheoesophageal groove lymph node (TGLN) metastasis with no increased adverse events.

In recent years, multimodal approaches are recommended in unresectable hepatocellular carcinoma (HCC), either as first-line or subsequent therapy. Some studies have shown that the combination of transarterial chemoembolization (TACE) and sorafenib or TACE and thermal ablation is superior to monotherapy. However, few data are available on patients with huge unresectable HCCs treated by TACE and sorafenib, with or without thermal ablation. Ying Wu et al. (Wu et al., 2020) retrospectively evaluate and compare the benefits of TACE and sorafenib with or without thermal ablation in the management of patients with huge unresectable HCCs. They provided a promising strategy of TACE-sorafenib-thermal ablation, which demonstrated extended long-term overall survival in patients with huge unresectable HCC, and this may be a better choice than TACE-sorafenib alone.

Esophageal cancer is one of the most common cancer types, with its most common distant metastatic site being the lung. Currently, population-based data regarding the proportion and prognosis of patients with esophageal cancer with lung metastases (ECLM) at the time of diagnosis is insufficient. Analyses conducted by Jida Guo et al. (Guo et al., 2021a) on Surveillance, Epidemiology, and End Results (SEER) database from 2010 to 2016 of ECLM indicated that age, number of extrapulmonary metastatic sites, treatment three factors as independent predictors for esophageal cancer-specific survival (CSS). Considering the factors that may predict the occurrence of lung metastasis at diagnosis, high-risk patients should undergo a 64-slice multidetector CT (MDCT) examination for small lung nodules screening. According to their findings, chemotherapy or chemoradiotherapy may represent the most advantageous treatments for patients with ECLM.

In conclusion, the “Chemoradiation Resistance in Cancer Therapy” research topic highlights the complex phenomenon of resistance to anticancer therapy in cancer cells. The recent research implies the need to continue improving our understanding into the fundamental mechanisms of chemoradiation resistance related to target mutations, tumor microenvironment, undiscovered genes and signaling pathways in cancers, with the aim of identifying relevant new biomarkers and to develop the strategies that can overcome chemoradiation resistance or improve patient care during chemo- and/or radiation therapy.
